# Critical Reflection into Action: Facilitating Conditions of Antiracist Action among White Youth in the Netherlands

**DOI:** 10.1007/s10964-025-02169-w

**Published:** 2025-03-26

**Authors:** Ymke de Bruijn

**Affiliations:** Department of Interdisciplinary Social Science, Faculty of Social and Behavioural Sciences, Utrecht University, Utrecht, Netherlands

**Keywords:** Critical consciousness, Antiracism, Youth, Ethnic-racial socialization, Intergroup contact

## Abstract

Critical reflection is a necessary yet not sufficient prerequisite for critical action, but little is known about conditions that facilitate associations between the two. This study investigates when associations between critical reflection on racism and antiracist action among White youth in the Netherlands are stronger, examining critical motivation, parental ethnic-racial socialization, and intergroup friendships as facilitating conditions. 338 17–21-years old White youth (*M* = 19.44, *SD* = 1.28, 73% female) participated in a survey study. The identified positive association between critical reflection and action was stronger for youth with higher levels of critical motivation. Parental ethnic-racial socialization and intergroup friendships, in contrast, did not moderate the association. Boosting critical motivation seems a promising avenue to fostering antiracism among White youth.

## Introduction

Antiracist action is crucial to counter racism, which manifests at individual and institutional levels in many societies, including the Netherlands (Kennedy et al., 2023; Thijssen et al., 2021). White Dutch youth are increasingly being informed about racism in society following a shift in the public debate (Ghorashi, 2020). Whereas this might increase their critical analysis of ethnic-racial inequalities in society, it does not necessarily translate into antiracist action, given that knowledge and behavior are not always correlated (Heberle et al., 2020). The current study therefore aims to examine when associations between knowledge (critical reflection) and behavior (antiracist action) are stronger, by assessing the role of critical motivation, parental ethnic-racial socialization, and intergroup friendships, and provides insights into how to support White youth to use their privileged positions to become an antiracist ally.

### Critical Consciousness

Racism is one of the most pressing problems many societies are facing, with detrimental effects on the physical, mental, and social health of those affected by it (Brondolo et al., 2012; Trent et al., 2019). Racism is often defined as a system in which beliefs, practices and policies based on race advantage groups that historically have had more power (i.e., White people in the context of the Europe) (Haeny et al., 2021). Biologically, racial groups do not exist, rather, they are socially constructed groups (Smedley & Smedley, 2005). These groups are not only constructed based on race as referring to physical characteristics, but also on cultural characteristics that have been racialized, as for example is the case for Muslims (Garner & Selod, 2015). As such, racism in Europe is not only based on beliefs of racial superiority, but also on beliefs of cultural superiority (Bratt, 2022), and the discourse in Europe speaks of racism on grounds of racial or ethnic origin (European Commission, 2020). Although race and ethnicity can be defined separately, with race stressing shared physical characteristics and ethnicity stressing shared ancestry, traditions, and cultural traits, they are often conceptualized as overlapping and used interchangeably (Bhopal, 2004). This is particularly true in the Western and Northern European context, where there generally is a “silence about race” (Jugert et al., 2022). As such, the present study applies a broad definition of racism, based not only on racial groups but also on ethnic and racialized groups, and uses ethnicity-race throughout.

In light of racism’s detrimental effects, the field of child development research has increasingly paid attention to effective approaches to reducing ethnic-racial prejudice among youth (Aboud et al., 2012; Beelmann & Heinemann, 2014). This work provides valuable insights into approaches to ensure that children are “not racist”. Racism, however, is not just an interpersonal phenomenon but also manifests at structural and institutional levels. In order to counter racism, therefore, “not being racist” is not sufficient, but antiracism is needed (Roberts & Rizzo, 2021). Antiracism involves awareness and understanding of racism at different levels and an active approach towards challenging it (Cooper et al., 2022).

Challenging racism can be studied through the lens of critical consciousness. Critical consciousness was first described by Paolo Freire, and historically represents marginalized people’s awareness of social inequality (reflection), perceived ability and responsibility to create change (motivation), and behavioral action taken to make this change (Watts et al., 2011). Critical motivation is often also referred to as political efficacy and conceptually aligns with sociopolitical control (Diemer et al., 2022). Although critical consciousness is grounded in work with adults, adolescence is a particularly relevant period for its development due to improved social cognitive skills (Brizio et al., 2015) that can be relevant to identifying injustice, and identity-formation processes which can involve commitment to an ideological stance (Marcia, 1980). Particularly in late adolescence, youth face developmental challenges of finding personal meaning and purpose in life, which can be accompanied by increased social awareness and activism (Barrett, 1996; Zarrett & Eccles, 2006). Critical consciousness is also considered a developmental asset for marginalized youth (Diemer et al., 2016), as it can have positive effects on their wellbeing (Maker Castro et al., 2022). Longitudinal research among adolescents shows that their critical consciousness is indeed still in development during this life phase (Pinedo et al., 2024; Wray-Lake et al., 2023).

Whereas research on adolescents’ critical consciousness has increased over the past few years, few studies have focused on White adolescents (Heberle et al., 2020). Although critical consciousness was originally conceptualized in the content of marginalized people, combating structural inequalities requires that all adolescents are able to reflect on, feel motivated to change, and act against these inequalities. True solidarity from privileged group members is therefore necessary (Freire, 2000). White adolescents are part of a social group that benefits from ethnic-racial inequalities in many societies, but can use their privileged positions and power in antiracist acts (Reason et al., 2005). White youth’s critical consciousness development is furthermore contextualized in a system of whiteness, which presents obstacles to this development that are unique to this group (Wray-Lake et al., 2023). To support White youth to use their potential, research on their development of critical consciousness is highly needed (Diemer et al., 2016; Rapa & Geldhof, 2020). Additionally, research among youth has mostly focused on their awareness of social inequality (i.e., critical reflection), yet centering the action component of critical consciousness is crucial to understand the potential of young people to bring about change in society (Diemer et al., 2021).

### Reflection Turned Into Action?

Critical reflection and action are theorized to develop reciprocally (Diemer et al., 2021; Watts et al., 2011). Some empirical work among marginalized youth indeed points towards positive associations between the two dimensions, so that youth who perceive more societal inequalities are engaged in more critical (Diemer & Rapa, 2016; Hope et al., 2020) and sociopolitical action (Banales et al., 2020). Similarly, youth themselves describe how their engagement in action depends on their knowledge on the issue at hand (Tyler et al., 2020). Theoretical models of White youth’s antiracism development likewise describe how an understanding of structural racism is essential (Hazelbaker et al., 2022; Woolverton & Marks, 2022). However, not all youth who are aware of societal inequality also behaviorally act to foster change: critical reflection and critical action are not always correlated (Heberle et al., 2020). Similarly, whereas some longitudinal studies support the idea that critical reflection precedes critical actions (Plummer et al., 2022), others do not find longitudinal associations between the dimensions of critical consciousness (Pinedo et al., 2024). This potential disconnect between reflection and action is also present in two out of five dispositions recognized in White American’s response to racism, which are marked by some form of cognitive awareness of racism accompanied by behavioral apathy (i.e., the rational and liberal disposition) (D’Andrea & Daniels, 1999). In two other dispositions, in contrast, cognitive awareness and understanding of racism do go together with engagement in behaviors aimed to foster change (D’Andrea & Daniels, 1999). The mismatch between reflection and act can be related to the principle-implementation gap (Dixon et al., 2017). The principle-implementation gap is referred to as an apparent paradox, describing how people who accept a certain principle (e.g., that equality is ideal) do not necessarily support, or even reject, interventions or policies aiming to achieve that same principle (e.g., affirmative actions) (Dixon et al., 2017). Research has demonstrated this gap in White Americans’, including youth, dispositions to ethnic-racial diversity and (in)equality (Hikido & Murray, 2016; Taylor & Parcel, 2019). Although critical reflection goes deeper than the mere acceptance of a principle, and critical action entails more than supporting policies, both “gaps” describe how beliefs and behavior do not necessarily align.

Reflection without action resembles so-called “armchair activists” (Diemer et al., 2016; Watts & Flanagan, 2007). This profile of critical consciousness not only is a loss of opportunity for society, as youth are aware of societal inequalities yet do not engage in actions to challenge them, it also links to direct negative outcomes for themselves such as lower life satisfaction and school engagement as compared to uncritical and inactive peers (Schwarzenthal et al., 2023). In particular for youth from more privileged social groups, such as White youth in White dominant societies, being an “armchair activist” furthermore seems to harmfully impact their capability to deal with academic challenges (Schwarzenthal et al., 2023). Especially if adolescents feel the need to act but do not actually do so, this might harm their well-being, as it limits their sense of competence which is essential for healthy psychological growth according to self-determination theory (Ryan & Deci, 2000). Understanding the conditions in which the association between critical reflection and critical action among White youth is strengthened is therefore essential both for society and for youth themselves.

### Facilitating Conditions

One potential facilitator of the association between critical reflection and critical action, is critical motivation (Watts & Flanagan, 2007). In particular, a lack of feelings of agency or perceived ability to create change may cause some youth to “get stuck” in critical reflection, whereas confidence in one’s abilities and feelings of empowerment may cause others to translate this reflection into action. This reasoning resonates with the Theory of Planned Behavior, which posits that one’s beliefs about their ability are an important determinant of behavioral intentions and, in turn, actual behaviors (Ajzen, 1991). Particularly, this theory proposes beliefs about ability to moderate the effect of behavioral intentions on actual behaviors (Montano & Kasprzyk, 2002). Empirical research on these proposed associations between critical consciousness domains is still somewhat limited (especially among White youth) (Heberle et al., 2020). Previous work using cluster analyses, however, did demonstrate that a combination of high critical reflection and critical motivation indeed goes together with more civic engagement (Christens et al., 2018). This is in line with previous qualitative work describing how youth experience beliefs that their actions would be ineffective or not taken seriously as barriers to engaging in critical action (Tyler et al., 2020), but needs further empirical replication. One empirical study testing moderation effects of critical motivation on the association between reflection and action among poor and working class youth of color in the U.S. did not find evidence for this hypothesis (Diemer & Rapa, 2016), but replications in a sample of youth in relatively privileged positions in society are currently lacking.

Additionally, White youth’s development of antiracism is thought to benefit from promotive contexts (Hazelbaker et al., 2022). These seem particularly relevant to overcome obstacles resulting from whiteness, such as limited opportunities to learn about racism in everyday life (Wray-Lake et al., 2023). Although parents are theorized to be important contributors to these contexts (Hazelbaker et al., 2022), empirical research on White parents’ influence on youth’s critical consciousness is very limited (Heberle et al., 2020). A recent exception shows that levels of critical reflection as well as action of White parents and their children are interrelated (Curran et al., 2023). The authors argue that modeling and socializing by White parents has the potential to teach their children ideas and actions that aim to challenge racism (Curran et al., 2023). Another recent exception confirms this idea, by demonstrating that White parents perceive their children to be more critically reflective after engaging in an anti-racist parenting intervention (Heberle et al., 2024). One type of socialization by parents that might be particularly important when it comes to White youth’s antiracism development is ethnic-racial socialization. Ethnic-racial socialization refers to the transmission of information regarding ethnicity and race from parents to their children, and has traditionally been characterized by four themes in work in minoritized families: cultural socialization (messages about one’s own ethnic-racial heritage or traditions), preparation for bias (messages preparing for discrimination), promotion of mistrust (messages warning about other ethnic-racial groups), and egalitarianism (messages emphasizing equality) (D. Hughes et al., 2006). Work on ethnic-racial socialization in White families has also investigated other themes of socialization, such as history of other groups (messages about the ethnic-racial heritage or traditions of others) and discrimination against other groups (Pahlke et al., 2012; Zucker & Patterson, 2018). Additionally, ethnic-racial socialization research in White families distinguishes between color-evasive (avoiding ethnicity- or race-related topics or downplaying the significance of ethnicity and race) versus color-conscious socialization practices (Abaied et al., 2022; Abaied & Perry, 2021).

Research in White families shows that parents often employ color-evasive socialization strategies in their general parenting approach (Vittrup, 2018) and in response to race-related situations (Abaied & Perry, 2021; Zucker & Patterson, 2018). If White parents do engage in color-conscious socialization, this has been hypothesized to contribute to children’s critical reflection (Hazelbaker et al., 2022). At the same time, White youth in “race conscious” contexts, who among other things have more frequent discussions about ethnicity or race with their parents, engage in more critical action than other White youth (Dull et al., 2022). What remains unclear, however, is whether parental ethnic-racial socialization can also strengthen *the association between* critical reflection and critical action. White people commonly experience anger, sadness, helplessness, guilt, and shame when being confronted with the realities and pervasiveness of racism in society (Spanierman & Heppner, 2004). Experiencing these emotions can be counterproductive, if experienced too strongly or not channeled properly (Spanierman & Cabrera, 2014). Having engaged in (more) frequent discussions about ethnicity and race with their parents might help White youth prepare to deal with potentially experiencing such emotions when engaging in critical reflection. These White youth might feel less overwhelmed or “paralyzed” by such emotions and might be better equipped to turn their knowledge into action. In this line of thought, particularly color-conscious forms of parental ethnic-racial socialization could play an important role.

At the same time, youth search for more autonomy from parents during adolescence, and friends become more important socialization agents (De Goede et al., 2009). Therefore, apart from parents shaping White adolescents’ ethnic-racial contexts, peers also play an important role (Dull et al., 2022). Contact with peers from different ethnic-racial backgrounds, in particular, may serve as a facilitating condition. Research based on intergroup contact theory has overwhelmingly demonstrated that positive intergroup contact, and particularly friendships, reduce group-based prejudice among privileged group members (Davies et al., 2011; Pettigrew & Tropp, 2006), even if not all optimal conditions (i.e., equal status, common goals, no intergroup competition, authority sanction) are met (Pettigrew et al., 2011). Additionally, having friends from underrepresented ethnic-racial groups is associated with stronger perceived injustice and discrimination among privileged group members (Bobowik et al., 2024; Carter et al., 2019) and as such sparks critical reflection. Intergroup contact can at the same time promote support for social change among privileged group members, as described in the Integrated Contact-Collective Action Model (Hässler et al., 2021). This model, based on intergroup contact theory and collective action research, describes that intergroup contact in general, but particularly frequent contact with intergroup friends, increases support for social change among advantaged groups (Hässler et al., 2021). Empirical work indeed supports this notion among advantaged groups based on migration status (Bobowik et al., 2024), ethnicity and sexual orientation (Hässler et al., 2021), and race (Selvanathan et al., 2018). The Integrated Contact-Collective Action Model also proposes that intergroup contact and privileged group members’ perceptions of the illegitimacy of group differences interact in predicting action, so that effects are strongest for those who both perceive these differences as unjust and engage in more frequent and intimate intergroup contact (Hässler et al., 2021). This raises the expectation that intergroup contact or friendships can impact *the association between* reflection and action. As experiencing support can help youth act (Diemer & Li, 2011; Tyler et al., 2020), intergroup friendships might provide just that for White youth in the context of antiracism. Intergroup friendships in particular might introduce them to contexts that provide more opportunities for antiracist action, and as such, function as opportunity structure for translating their knowledge into action (Watts & Flanagan, 2007).

### Research Context

The Netherlands provides an interesting context for research on critical consciousness among White youth. The Dutch population is highly diverse in terms of its ethnic-racial background, given that more than a quarter of the population has a migration background (first or second generation) of which two-thirds are from outside of Europe (CBS, 2024b). Turkish, Moroccan, Surinamese, Indonesian, and Caribbean backgrounds are most common (CBS, 2024b). Experiences of racism are common among these people. For example, almost a third of people born in the Netherlands with parents born in Suriname, Morocco, or the Dutch Caribbean reported to have been discriminated against based on their race or skin color in 2023 (CBS, 2024a). In the same year, Geert Wilders’ radical right-wing populist party Party for Freedom, characterized by extreme Islamophobic and anti-immigration sentiments (Vossen, 2011), became the biggest political party during national elections (van Holsteyn & Irwin, 2024). At the same time, following the death of George Floyd and Black Lives Matter protests in the United States, the antiracist movement in the Netherlands gained attention in the public debate (Ghorashi, 2020). Additionally, reports increasingly acknowledge institutional racism in the Netherlands, for example at the labor and housing market (Felten et al., 2021), and at the national government (Omlo et al., 2022; Witkamp et al., 2024). Furthermore, the Dutch role in slavery is increasingly acknowledged, with developments in apologies being made by the royal family, local governments, and the national government (Hendriks et al., 2024). These recent developments have caused an increase in attention for racism in the Netherlands. This is huge shift, given that racism in the Netherlands previously has hardly been acknowledged in the public and societal discourse (Ghorashi, 2014; Weiner, 2014). In fact, denial of racism is described as one of the characterizing features of Dutch racism (Essed & Hoving, 2014).

This denial of racism and race-mute characteristic of the Netherlands is also reflected in the racialized terms to refer to different groups of people. For example, “allochtoon” was used until 2016 (De Ree, 2016) which formally referred to people born or with at least one parent born outside of the Netherlands but in practice was used to refer “non-Western” ethnic-racial groups (Essed & Trienekens, 2008). This term was later replaced with “migration background”, which is similarly racialized in the European context (Moffitt & Juang, 2019) and used to avoid naming ethnic-racial groups explicitly (Jugert et al., 2022; Vietze et al., 2023). As such, “migration background” is often used as a racialized comparison to White, non-immigrant Europeans (Moffitt & Juang, 2019). Whereas White traditionally refers to a skin color, it is conflated with European (Ammaturo, 2019; Begum, 2023) and Dutch identities (de Bruijn et al., 2024b; Essed & Trienekens, 2008). Whiteness is furthermore characterized by “White experience”, being part of the privileged or dominant group (Lewis, 2004), and the concept of whiteness has over time excluded non-European White identities (Bonnett, 1998), excluding people with light skin tones who have migration backgrounds from outside of Europe.

Because of the denial of racism that has been common in the Netherlands for long (Essed & Hoving, 2014), most White Dutch youth have for long been exposed to color-evasive social norms in schools (Sijpenhof, 2020), and frequently also at home (de Bruijn et al., 2024a; Mesman et al., 2022). Through the rise in attention for the subject of racism in the public discourse, White Dutch youth are likely to become more informed and knowledgeable about the subject, potentially sparking their critical reflection. Nonetheless, this does not mean that all of them will also become more active in challenging racism. Understanding when White Dutch youth translate critical reflection into antiracist action is key to raising new generations who are not only knowledgeable of societal inequalities, but who also engage in activities to confront them.

Previous research on Dutch youth has looked into their ethnic-racial relations among Dutch youth. Particularly, a considerable amount of work has documented ethnic-racial attitudes and prejudices among ethnic-racial majority (e.g., de Bruijn et al., 2020; Thijs et al., 2023) and minority members (Pektas et al., 2023), as well as their intergroup contact levels and ethnic-racial identities (e.g., Fortuin et al., 2014; Verkuyten, 2001). Work examining their tendencies towards *anti*racism, however, is currently lacking.

## Current Study

Critical consciousness research so far has mostly focused on youth of color, and on the reflection component of critical consciousness. As such, little is known about White youth’s pathways to critical antiracist action. The present study examines the association between critical reflection on racism and antiracist action among White youth in the Netherlands. In particular, the present study aims to identify when associations between these constructs are stronger, by examining critical motivation, parental ethnic-racial socialization, and intergroup friendships as potential moderators. It is expected that associations between critical reflection on racism and antiracist action among White youth in the Netherlands are stronger if youth have a stronger sense of critical motivation (Hypothesis 1), have experienced more color-conscious parental ethnic-racial socialization (Hypothesis 2), and have more intergroup friendships (Hypothesis 3).

## Methods

An overview of measures, data, and syntax can be found in an open repository: https://osf.io/g4r8x. Preregistration of the study and hypotheses can be found through: https://osf.io/unz59.

### Sample and procedures

Adolescents were recruited through youth organizations and student associations, snowballing, the researchers’ network, face-to-face recruitment at campus, and social media advertisements. Adolescents could participate if they (1) were between 17 and 21 years old and (2) self-identified as White^1^. After having read the information letter and digitally giving consent, participants completed an online survey which took approximately 20–30 min. Participants first answered questions on their background characteristics, after which they completed the other measures. After having completed the questionnaire, participants could opt to receive a digital gift card of €5.

A total of 403 adolescents participated in the study. After removing 40 incomplete responses (meaning that they only filled out background characteristics), as well as 25 participants who apart from identifying as White indicated to also identify with another ethnic-racial background, a total of 338 adolescents were retained for analyses. Participants were between 17 and 21 years old (*M* = 19.44, *SD* = 1.28). Most of the participants identified as female (73%), 20% of the participants identified as male, and 4% of the participants identified as non-binary. Others reported another gender identity (2%), or did not want to report their gender identity (1%). Most participants (98%) were born in the Netherlands, as were their parents (95% of mothers and 97% of fathers), with (one of) whom 67% of the participants were living. The majority of the participants were still in school (94%), of which 10% was in high school, 7% was in secondary vocational education (MBO), and 82% was in tertiary higher education (HBO and WO).

### Measures

#### Critical reflection

Critical reflection was measured with an adapted version of the measure as used by Bañales et al. (2020). The original measure presented adolescents with 13 statements addressing causal attributions for the educational achievement gap between White and Black students, of which five referred to structural attributions and eight referred to individual attributions. The original description of the achievement gap as well as the attributions were evaluated by the researcher and three independent experts in the field of educational inequalities in the Netherlands, and based on their input adaptions were made to fit the Dutch context (the full adapted version can be found at https://osf.io/g4r8x). In the adapted version, adolescents were presented with 18 statements addressing causal attributions for the educational achievement gap between young people with a migration background and without a migration background. This includes 12 of the original items, with small adaptations for some (e.g., “are often discouraged to pursue higher levels of education” instead of “are often discouraged from being in Honors or AP classes”). One of the original items was deleted, as the experts who reviewed the scale agreed that it did not reflect a sentiment from the Dutch context (“Black students aren’t as good at subjects like math and science as White students”). Additionally, six items were added based on expert input, referring to students’ home environment (i.e., having a quite room to study, getting help with schoolwork at home), peer environment (i.e., being less encouraged by friends to work on school), language (i.e., speaking Dutch less well, learning Dutch less well at home), and motivation (i.e., being less motivated for school). Items were answered on a five point Likert scale, ranging from strongly disagree (1) to strongly agree (5). Factor analyses were conducted to determine the structure of the scale (see supplementary materials 1). Critical reflection is conceptualized as the structural attributions subscale. Results from the factor analyses indicated that 5 items loaded on the structural attributions subscale (i.e., all five adapted items from the original structural attributions subscale), and thus will be used in further analyses. Cronbach’s alpha for the structural attributions subscale was 0.79.

#### Critical action

Critical action was measured with an adapted version of the Anti-Racism Action Scale (Aldana et al., 2019). The original measure presented youth with 18 items reflecting different types of antiracist behaviors (i.e., 7 interpersonal, 4 communal, 7 political). A pilot study was conducted to evaluate whether this measure, that was developed by American youth, also adequately reflects antiracism among Dutch youth. A group of 10 young adults in the Netherlands were interviewed, during which they were asked to (1) evaluate whether they recognized the items from the original scale as reflecting antiracist behaviors in the Netherlands, (2) indicate whether any important behaviors were missing, and (3) indicate whether they identified other improvements. Based on these interview results, all original items were deemed appropriate for the Dutch context, although some additional examples were added and some formulations were slightly changed (the full adapted version can be found at https://osf.io/g4r8x). Additionally, five items were added to reflect antiracist behaviors recognized as relevant to the Dutch context (i.e., being active for a (youth) political party that addresses racism, speaking out against racism through social media, donating money to an organization that combats racism, signing a petition against racism, and reporting (online) racism at discrimination reporting points). For all items, participants were asked to indicate whether they had ever engaged in them (0 = no, 1 = yes). Factor analyses were conducted to determine the structure of the scale (see supplementary materials 2). A one-factor solution was sought to be used in the main analyses, as no specific hypotheses were formulated about subscales or forms of critical action. An acceptable one-factor model fit was found with 17 of the 23 items. Cronbach’s alpha for this total scale was 0.81. Nonetheless, subfactors were assumed and were planned to be used in exploratory analyses. Therefore, exploratory factor analyses were also run, but no multi-factor model could reliably be fit to the data (see supplementary materials 2).

#### Critical motivation

Critical motivation was measured with the Motivation Subscale of the Short Critical Consciousness Scale (Diemer et al., 2022). This questionnaire presents participants with four statements reflecting their motivation to work towards equality, to be answered on a six point Likert scale, ranging from strongly disagree (1) to strongly agree (6). An example item is “It is important to correct social and economic inequality”. The four items were used as indicators of the latent variable. Cronbach’s alpha for this scale was 0.69.

#### Parental ethnic-racial socialization

Parental ethnic-racial socialization (ERS) was measured with the Parental Racial-Ethnic Socialization Behaviors measure (D. Hughes & Chen, 1997; D. Hughes & Johnson, 2001) as adapted for White families (Pahlke et al., 2012). Additionally, the phrasing of the scale was changed in order to be used as a self-report measure among youth. This scale consists of 25 items tapping into different types of ethnic-racial socialization behaviors and messages. The 25 items have been used in different numbers of subscales in prior research (i.e., four subscales – egalitarianism, history of other groups, discrimination against other groups, preparation for bias (Pahlke et al., 2012) and six subscales – adding the subscales group difference and general discrimination (Zucker & Patterson, 2018)). Therefore, factor analyses were conducted to determine the structure of the scale in the present study (see supplementary materials 3). For the present study, three factors resulting from the factor analyses seemed theoretically most relevant (reflecting egalitarianism, history of other groups and discrimination against other groups), consisting of 6, 4 and 8 items respectively. Cronbach’s alpha for these subscales were 0.86, 0.86 and 0.90, respectively.

#### Intergroup friendships

Intergroup friendships were measured by asking youth to report how many of their friends are of Color, on a five point Likert scale ranging from (almost) none (1) to almost all (5). Additionally, youth were asked to report how much time they spend with their friends of color on a similar scale ranging from never (1) to everyday (5). For this study, scores from the second question were used as this suggests behavioral engagement (Davies et al., 2011).

### Analyses

First, descriptive statistics of and bivariate correlations between the main variables are assessed in SPSS 29.0 (using simple mean scores of main variables, because the mean structure of latent variables was created by setting latent means to zero). After, structural equation modeling (SEM) is used in Mplus. Latent variables were created for critical reflection, critical action, critical motivation and parental ethnic-racial socialization dimensions based on the factor analyses (see Supplementary Materials). Missing data were addressed under Full Information Maximum Likelihood conditions (FIML). For each model, after fitting the measurement model, the first step was fitting the structural model with main effects only, and in the second step, if applicable, the interaction was added. Model 1 examines the association between latent variables critical reflection and critical action. Model 2 models critical action on critical reflection, critical moderation, and the interaction between critical reflection and critical moderation. Model 3a, 3b, and 3c model critical action on critical reflection, parental ethnic-racial socialization dimensions, and the interaction between parental ethnic-racial socialization dimensions and critical reflection. The three forms of parental ethnic-racial socialization are examined as separate moderators in separate models^2^ (3a for egalitarianism, 3b for history of other groups and 3c for discrimination against other groups, respectively). Model 4 models critical action on critical reflection, intergroup friendship, and the interaction between critical reflection and intergroup friendship. As such, each model tested only one moderator at the time, to avoid convergence issues related to exceeding the maximum number of integration points. Indicators of critical reflection and moderator latent variables were standardized before creating interaction terms. Significant interactions are followed up by testing simple slopes at levels of the mean and +/− 1 SD from the mean of the moderator.

## Results

### Descriptive Statistics

Descriptives statistics of and bivariate correlations between main variables can be found in Table 1. Correlations show significant positive associations between the three dimensions of critical consciousness, as well as between the three subscales of parental ERS. Critical reflection is additionally only positively related to parental ERS about discrimination against other groups, whereas critical action and critical motivation are positively related to parental ERS about discrimination against other groups and egalitarianism. Lastly, intergroup friendships are positively related to critical action and the three subscales of parental ERS.

**Table 1:** Descriptive statistics of scales and bivariate correlations

	*n*	*M*	*SD*	1	2	3	4	5	6
1. Critical reflection	321	3.68	0.70						
2. Critical action	311	0.31	0.20	0.45***					
3. Critical motivation	338	5.00	0.62	0.43***	0.50***				
4. ERS egalitarianism	303	2.88	0.92	0.07	0.14*	0.22***			
5. ERS history	303	2.33	0.90	0.02	0.07	0.10	0.55***		
6. ERS discrimination	303	2.97	0.85	0.16**	0.17**	0.22***	0.70***	0.65***	
7. Intergroup friendships	303	2.97	1.11	0.10	0.23***	0.10	0.15**	0.13*	0.12*

*p <0.05, **p <0.01, ***p <0.001

### Structural Equation Modelling

**Fig. 1:**
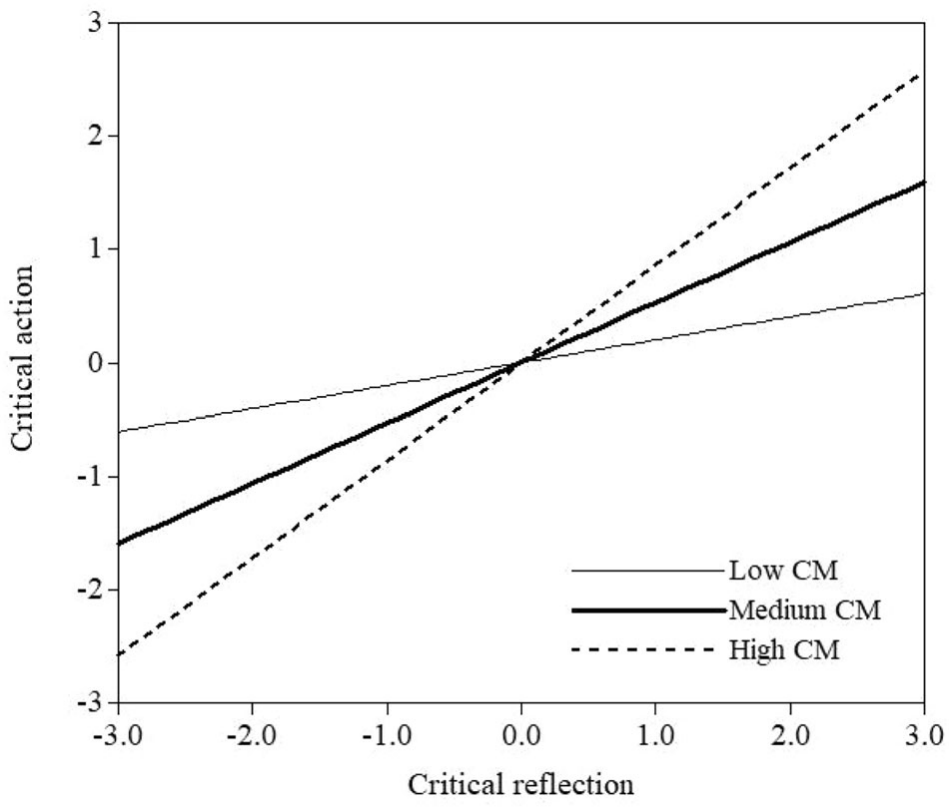
Moderation effect of critical motivation (CM) on the association between critical reflection and critical action

Results from structural equation modelling are shown in Table 2. Results from Model 1 show that there is a significant positive association between critical reflection and critical action. Results from Model 2 additionally show that there is a significant association between critical motivation and critical action, and that critical motivation significantly moderates the association between critical reflection and critical action. The simple slopes at the mean level of critical moderation and 1 SD below and above the mean are displayed in Fig. 1, and indicate that the association between critical action and critical reflection is stronger for those higher in critical motivation. Results from Models 3a-c show that none of the dimensions of parental ERS significantly relate to critical action, and that none of them emerge as a significant moderator. Lastly, results from Model 4 show that there is a significant positive association between intergroup friendships and critical action, but that intergroup friendships do not moderate the association between critical reflection and critical action.

**Table 2:** Results from structural equation models

	Standardized path coefficient [95% CI]		*n*	*R*^*2*^	χ^2^	df	RMSEA	SRMR	CFI	TLI
*Model 1 (step 1)*			321	0.34	358.11***	208	0.05	0.11	0.91	0.90
CR → CA	0.58***	[0.48, 0.69]								
*Model 2 (step 1)*			338	0.57	508.34***	296	0.05	0.10	0.91	0.91
CR → CA	0.24**	[0.09, 0.39]								
CM → CA	0.59***	[0.45, 0.74]								
*Model 2 (step 2)*			338	0.66						
CR → CA	0.23**	[0.05, 0.40]								
CM → CA	0.65***	[0.49, 0.81]								
CR*CM → CA	0.09*	[0.01, 0.17]								
*Model 3a (step 1)*			321	0.35	559.41***	347	0.04	0.11	0.90	0.89
CR → CA	0.57***	[0.47, 0.68]								
ERS egalitarianism → CA	0.09	[−0.02, 0.20]								
*Model 3a (step 2)*			321	0.38						
CR → CA	0.60***	[0.49, 0.72]								
ERS egalitarianism → CA	0.11	[<−0.01, 0.23]								
CR*ERS egalitarianism → CA	0.01	[−0.13, 0.14]								
*Model 3b (step 1)*			321	0.34	0.456.47***	296	0.04	0.10	0.92	0.91
CR → CA	0.58***	[0.48, 0.69]								
ERS history → CA	0.07	[−0.05, 0.18]								
*Model 3b (step 2)*			321	0.40						
CR → CA	0.62***	[0.51, 0.73]								
ERS history → CA	0.05	[−0.07, 0.17]								
CR*ERS history → CA	0.12	[−0.02, 0.25]								
*Model 3c (step 1)*			321	0.34	630.09***	402	0.04	0.10	0.90	0.88
CR → CA	0.57***	[0.46, 0.68]								
ERS discrimination → CA	0.06	[−0.05, 0.18]								
*Model 3c (step 2)*			321	0.38						
CR → CA	0.59***	[0.48, 0.71]								
ERS discrimination → CA	0.10	[−0.03, 0.22]								
CR*ERS discrimination → CA	−0.02	[−0.15, 0.11]								
*Model 4 (step 1)*			321	0.37	408.04***	228	0.05	0.11	0.91	0.90
CR → CA	0.56***	[0.46, 0.71]								
Intergroup friendships → CA	0.17**	[0.07, 0.32]								
*Model 4 (step 2)*			321	0.41						
CR → CA	0.59***	[0.48, 0.71]								
Intergroup friendships → CA	0.19**	[0.08, 0.31]								
CR*Intergroup friendships → CA	−0.02	[−0.14, 0.10]								

*CR* critical reflection, *CA* critical action, *CM* critical motivation, *ERS* ethnic-racial socialization

*p <0.05, **p <0.01, ***p <0.001

## Discussion

Two important caveats have been identified in the literature on (youth’s) critical consciousness, namely the limited focus on critical action and youth in privileged positions (Diemer et al., 2021; Heberle et al., 2020). Additionally, although critical reflection and critical action are theorized to develop reciprocally (Diemer et al., 2021; Watts et al., 2011), not all empirical work finds associations between the two dimensions of critical consciousness (Heberle et al., 2020; Pinedo et al., 2024). As such, this raises the question of what conditions can facilitate associations between critical reflection and critical action. Therefore, the present study examined whether the association between critical reflection on racism and antiracist action among White youth in the Netherlands is moderated by critical motivation, parental ethnic-racial socialization, and intergroup friendships. Results show that the positive association between critical reflection on racism and antiracist action was stronger for individuals with higher levels of critical motivation, whereas experiences with parental ethnic-racial socialization and intergroup friendships did not impact the association between critical reflection and critical action.

The positive association between critical reflection and critical action that was found in the present study aligns with the conceptualization of critical consciousness dimensions developing reciprocally (Diemer et al., 2021; Watts et al., 2011), and with previous empirical work establishing a positive association among marginalized youth (Banales et al., 2020; Diemer & Rapa, 2016). This study extends this insight to White youth in a White dominant society, aligning with theoretical models of antiracism development among White youth that emphasize the relation between understanding racism and developing antiracism (Hazelbaker et al., 2022; Woolverton & Marks, 2022). Due to the cross-sectional nature of the study, results cannot speak to the directionality of this association. It therefore remains open for further investigation whether critical reflection precedes critical action as suggested in these developmental models.

The main aim of the present study was to understand the conditions under which the association between critical reflection and critical action in the domain of racism among White youth would be strengthened. In line with the hypothesis, results indicate that critical motivation is one of the facilitators of this association. In particular, the association between critical reflection and critical action was stronger for youth who reported higher levels of critical motivation. This finding aligns with and provides important empirical evidence for the idea that sense of agency is an empowering factor that helps youth who critically analyze social problems to take action (Watts & Flanagan, 2007). These youth are thus less at risk for becoming “armchair activists”, characterized by reflection without action (Diemer et al., 2016; Watts & Flanagan, 2007). Strengthening youth’s critical motivation could be an avenue to support critically reflective youth to engage in critical action, and thereby contribute to social change. On the societal level, this involves taking youth seriously (Tyler et al., 2020), by structurally including them in decision making processes, for which a new strategy is currently in development in the Netherlands (Rijksoverheid, 2024). On the individual level, values-affirmation interventions in which youth are encouraged to reflect on why certain values are important to them might help to raise critical motivation, but further research in this area is needed (Rapa et al., 2020).

In contrast to expectations, the present study did not find parental ethnic-racial socialization to moderate the association between critical reflection on racism and antiracist action among White Dutch youth. Previous research established that White parents play a role in shaping their children’s critical reflection and action (Curran et al., 2023; Dull et al., 2022), but the results from the present study suggest that parental ethnic-racial socialization does not impact *the association between* critical reflection and action. At the same time, the present study did not find consistent and robust associations between parental ethnic-racial socialization dimensions and critical reflection and action. Although parental socialization is seen as an important part of providing a promotive context for White youth to develop antiracism (Hazelbaker et al., 2022), parental socialization can take many shapes and forms. Results from the present study by no means imply that parental socialization does not have a role in shaping White youth’s critical consciousness at all. Rather, it is key to further investigate the different effects of various types of socialization messages at different developmental stages in order to identify facilitating mechanisms in youth’s critical consciousness development. Results should at the same time be interpreted with caution, as the identified dimensions of parental ethnic-racial socialization in the present study did not fit the data very well. Given that measures of parental ethnic-racial socialization are mostly developed and used in the U.S. (Yasui, 2015), there is a high need for the development of psychometrically sound measures to be used in other sociocultural and -historical context such as the Netherlands.

As a last potential moderator, it was hypothesized that spending more time with friends of color would strengthen the association between critical reflection on racism and critical action among White Dutch youth. Results, however, did not confirm this hypothesis. Spending time with friends of color was related to more critical action, but did not moderate *the association between* critical reflection and critical action. The fact that youth who spent more time with friends of color were engaged in more critical action aligns with previous research finding that intergroup contact promotes support for social change (Bobowik et al., 2024; Hässler et al., 2021). Whereas intergroup friendships did not seem to function as providing opportunities for youth to translate their knowledge into action (Watts & Flanagan, 2007), they might function as opportunities to engage in critical action, irrespective of one’s level of critical reflection. Potentially, youth who spend more time with friends of color experience more support, which can help youth act (Diemer & Li, 2011; Tyler et al., 2020). Longitudinal work is needed to disentangle how intergroup friendships impact different dimensions of critical consciousness over time, as engaging in critical action without critical reflection might actually spark reflection (Diemer et al., 2021). From the present cross-sectional results, it seems that spending time with friends of color relates to White youth’s behavior (critical action) more so than to their cognitive analysis of society (reflection). Perhaps other dimensions of intergroup friendships (e.g., self-disclosure, closeness) (Davies et al., 2011) are more relevant for critical reflection and/or the association with critical action. Additionally, the present study did not examine the quality of friendships, the extent to which key conditions of intergroup contact as outlined by Allport were met (Pettigrew & Tropp, 2006), or the extent to which other moderating factors of the effect of intergroup contact according to the Integrated Contact-Collective Action Model for advantaged groups were present (Hässler et al., 2021). It might be that these indicators of the (context of the) relationship influence intergroup friendships’ effects on critical consciousness development.

There are some limitations to the present study that should be taken into account. First of all, most of the measures have not been used in the Dutch context before. The measures of critical reflection and critical action were therefore slightly adapted based on expert insights and youth perceptions gathered in a pilot study. Although this strengthens the ecological validity of these measures, the exploratory nature of the use of these instruments needs to be taken into account when interpreting the results, and psychometric properties of these instruments in this specific context need further investigation. As is the case for most existing quantitative measures of critical reflection, the measure in the present study only captures youth’s critical reflection at that specific timepoint, even though critical reflection is an ongoing process (Diemer et al., 2015). Furthermore, the critical reflection measure only captures reflection on structural attributions to ethnic-racial inequalities in a specific domain, namely education. Although this is a domain that is relatively salient in the lives of the participants, who were mostly still in schools themselves, it does not necessarily translate to their critical reflection on racism in other domains. All measures furthermore involved self-report by participants, which heightens the risk of social desirability.

Secondly, the factor analyses that were conducted to investigate the underlying structure of the constructs and measures revealed that the dimensions of parental ethnic-racial socialization identified and used in the present study did not fit the data very well. The factor analyses furthermore showed that no reliable subscales of the antiracist action measure could be identified (Aldana et al., 2019). Although no specific hypotheses had been formulated for subtypes of antiracist action, it would be highly relevant to zoom in on different types of actions in future research, albeit in an exploratory way. It is likely that youth experience different barriers for engaging in this wide range of antiracist actions, from (inter)personal actions to public actions aimed at political change (Nelson et al., 2011), and thus different facilitating conditions might be at play.

Thirdly, there are some limitations to the generalizability of results, as women and highly educated youth were overrepresented in the sample. Future research should explore whether similar associations between dimensions of critical consciousness are found among youth with other social group memberships. Other possibly intersecting social identities of participants were not taken into account in the present study. As these can influence their sense and experience of privilege versus marginalization, future research should apply an intersectional lens and look into dimensions of critical consciousness along multiple lines of social group membership (Wray-Lake et al., 2023). Additionally, generalizability might be constraint to the specific topic of racism, and future work is needed to explore whether similar patterns are observed when critical consciousness is investigated along other lines of social inequalities. In this light is it also important to note that the measure of critical motivation used in the present study is a general measure, not specifically tailored to racism, whereas the measures of critical reflection and action are. Critical motivation furthermore encompasses both internal and external processes (Diemer & Rapa, 2016), which were not distinguished in the present study but open up additional room for future endeavors.

Lastly, although it is a strength of the current study that it focusses on two highly relevant socialization agents in youth’s environment (i.e., parents and peers), it was not examined how they and their role in shaping youth’s critical consciousness interact. Future research is needed to disentangle how different socialization agents, such as parents and peers, but also social media (Choi et al., 2023), can play a role in shaping the different dimensions of critical consciousness and their interplay, in line with an ecological approach to socialization (D. L. Hughes et al., 2016).

## Conclusion

Most of the work on critical consciousness has focused on how to increase youth’s critical analysis of societal inequalities. Although this is an important endeavor, not all youth who engage in this critical analysis also engage in actions against societal inequalities that can actually create social change. Among White Dutch youth, one factor was identified that facilitated stronger associations between critical reflection and critical action in the context of racism: critical motivation. Boosting youth’s critical motivation thus seems a promising avenue in order to foster antiracism that is based on a critical analysis of ethnic-racial inequalities in society among youth in a privileged position. These insights can spark new research on how to help White youth use their potential to contribute to a fairer society.

## Supplementary information

Supplementary Materials

## Footnotes

1 Participants were asked whether they identified as White, with the explanation that White in this study refers to people with a light skin color of European descent.

2 Please note that this a deviation from the preregistration, where it was stated that the dimensions of parental ethnic-racial socialization would be tested as moderators within one model. Including them in one model, however, resulted in convergence issues due to the number of integration points needed.
