# Supplementary material for: Critical Reflection into Action: Facilitating Conditions of Antiracist Action among White Youth in the Netherlands
Source: J Youth Adolesc. 2025 Mar 26;54(8):1902–16. doi: 10.1007/s10964-025-02169-w (PMC12331876; doi:10.1007/s10964-025-02169-w)
Supplement: Supplementary file 1 — Supplementary Materials [file 10964_2025_2169_MOESM1_ESM.pdf]

Supplementary Materials to “Critical Reflection into Action: Facilitating Conditions of Antiracist Action among White Youth in the Netherlands”

Supplementary Materials 1: EFA on critical reflection measure

An exploratory factor analysis (EFA) was run on the critical reflection measure that was adapted from the measure by Bañales et al. (2020). The EFA was conducted using Mplus 8.10, allowing for correlations between factors by using Geoming rotating and addressing missing data under Full Information Maximum Likelihood conditions (FIML). Between 2 and 6 factor-solutions were evaluated. The number of factors is determined based on eigenvalues, model fit indices, and interpretability of the factors. Furthermore, factors should have at least three indicators and indicators were considered salient if factor loadings were above .40. In case of cross-loadings, items were retained to the factor it loads .40 or higher on. If items loaded above .40 on multiple items, they were deleted in order to retain a simple structure.

Eigenvalues pointed in the direction of a 3-factor solution, with eigenvalues of 5.78, 3.04 and 1.65 for the first three factors and below 1 for the factors following. Model fit indices (as provided in Table S1) indicated that the 2-factor solution did not provide a good fit (i.e., RMSEA >.08, CFI and TLI <.90). From the 3-factor solution onward, model fit indices did indicate an acceptable or good fit to the data. Additionally, the 4-, 5-, and 6-factor solution all only had three factors with a minimum of three indicators loading .40 or higher (after removing indicators loading .40 or higher on multiple indicators and assigning other cross-loading indicators to the factor they load highest on). Therefore, the 3-factor solution was retained.

Table S1. Model fit indices for 2 to 6 factor solutions on critical reflection measure

	χ^2 (df)	*p*	RMSEA	SRMR	CFI	TLI
2-factor	421.55 (118)	<.001	0.09	0.06	0.88	0.84
3-factor	200.59 (102)	<.001	0.06	0.03	0.96	0.94
4-factor	151.81 (87)	<.001	0.05	0.02	0.97	0.95
5-factor	104.48 (73)	.001	0.04	0.02	0.99	0.97
6-factor	76.19 (60)	.078	0.03	0.02	0.99	0.98

Factor loadings for the 3-factor solution are presented in Table S2. The factor loadings show that factor 1 reflects the structural attributions subscale, as all (adapted) items from the original structural attributions subscale load on this factor. Factor 2 reflects the individual attributions subscale, as all (adapted) items from the original individual attributions subscale load on this factor, as well as two of the newly added items (referring to youth's motivation and encouragement by peers). Four other new items seem to for a separate factor, factor 3, reflecting attributions related to language and home environment. In the present study, factor 1, reflecting structural attributions, will be used as a measure of critical reflection.

Table S2. Factor loadings for the 3-factor solution of critical reflection (EFA)

	F1	F2	F3
Young people with a migration background don't have as many advantages as young people without a migration background that help them at school. (S)	**.53**	.04	-.05
Because of their background, young people with a migration background often are advised to go to lower levels of education than young people without a migration background. (S)	**.83**	-.06	.01
Young people without a migration background often go to better schools than young people with a migration background. (S)	**.46**	-.04	.14
Teachers expect young people without a migration background to do better in school than young people with a migration background. (S)	**.73**	.01	-.02
Young people with a migration background are often discouraged to pursue higher levels of education. (S)	**.70**	-.06	-.01
Young people with a migration background think it's not cool to spend a lot time on school work. (I)	-.06	**.76**	-.02
Young people with a migration background think school success is less important than young people without a migration background. (I)	-.05	**.82**	-.05
Young people with a migration background don't work as hard at school as young people without a migration background. (I)	-.02	**.83**	.00
For young people with a migration background, doing well in school is not important to become successful. (I)	-.01	**.73**	-.03
Young people without a migration background are smarter than young people with a migration background. (I)	-.12	**.52**	.05
Young people with a migration background do not spend a lot of time on school work.(I)	.07	**.84**	.01
Young people with a migration background are better are at other things than at school work. (I)	.16	**.64**	.04
Young people with a migration background more often do not have a quiet place to study than young people without a migration background. (N)[a]	.34	.02	**.45**
Young people with a migration background do not speak Dutch at the same level as young people without a migration background. (N)	.00	.01	**.71**
Young people with a migration background are less motivated for school than young people without a migration background. (N)	.00	**.83**	-.05
Young people with a migration background are not encouraged by their friends to do well at school as much as young people without a migration background. (N)	.15	**.50**	.23
Young people with a migration background get less support in school work at home than young people without a migration background. (N)	.12	.00	**.63**
Young people with a migration background are not taught Dutch as well at home as young people without a migration background. (N)	-.01	-.21	**.81**

Note. F = factor. (S) = (adapted) item from structural subscale. (I) = (adapted) item from individual subscale. (N) = new item. **Bold** = indicator of this factor.

Supplementary Materials 2: CFA and EFA on critical action

A 1-factor confirmatory factor analysis (CFA) was run on the critical action measure that was adapted from the measure by Aldana et al. (2019). The CFA was conducted using Mplus 8.10 using a categorical approach (Weighted Least Squares, Mean and Variance estimator; WLSMV) due to binary nature of the items. When converging the model, warnings indicated that due to low variability in some of the variables, bivariate tables included empty cells. Therefore, variables with lower than 5% of responses in one of the answer categories were excluded from the analyses (n = 5). Most model fit indices indicated the 1-factor solution on the 18 remaining items to be an acceptable/good fit, $\chi^2(135) = 279.80$, $p < .001$, RMSEA = 0.06, CFI = 0.92, TLI = 0.91, with the exception of the SRMR = 0.13. One of the items did not meet the pre-established cut-off of standardized factor loadings higher than .40, and thus an additional CFA was run excluding this item. Again, most model fit indices indicated this one-factor solution on the remaining 17 items to be an acceptable/good fit, $\chi^2(119) = 260.68$, $p < .001$, RMSEA = 0.06, CFI = 0.92, TLI = 0.91, with the exception of the SRMR = 0.13. Therefore, this 1-factor solution was used in further main analyses. Standardized factor loadings are reported in Table S3.

Additionally, an EFA was run on the critical action measure (excluding the low frequency variables) to explore whether meaningful subscales of acritical action could be identified to be included in exploratory analyses. The EFA was conducted using Mplus 8.10, allowing for correlations between factors by using Geoming rotating and using the Weighted Least Squares, Mean and Variance adjusted estimator to because of the binary nature of items. Between 1 and 6 factor-solutions were evaluated[1]. The number of factors is again determined based on eigenvalues, model fit indices, and interpretability of the factors. Furthermore, factors should have at least three indicators and indicators were considered salient if factor loadings were above .40. In case of cross-loadings, items were retained to the factor it loads .40 or higher on. If items loaded above .40 on multiple items, they were deleted in order to retain a simple structure.

---

[1] Please note that this is a deviation from the preregistration. It was preregistered that between 1 and 7 factor-solutions would be evaluated. However, given that the number of items dropped to 18 due to low frequencies, a 7-factor solution no longer was appropriate.

Table S3. Factor loadings for 1-factor solution of critical action (CFA)

	F1
Challenged or checked a friend who uses a racist slur or makes a racist joke.	**.55**
Challenged or checked a family member who uses a racist slur or makes a racist joke.	**.43**
Challenged or checked an adult who uses a racist slur or makes a racist joke who is not a family member (i.e. parent's friend, coach, boss, teacher, etc.).	**.50**
Defended a friend who is the target or a racist slur or joke.	**.40**
Defended a stranger who is the target of a racist slur of joke.	**.47**
Talked with friends about issues of ethnicity, heritage and/or discrimination.	**.58**
Attended a meeting on an issue related to ethnicity, heritage and/or discrimination.	**.76**
Joined a club or group working on issues related to ethnicity, heritage and/or discrimination.	**.71**
Tried to get into a leadership role or committee working on issues related to ethnicity, heritage and/or discrimination (e.g., student council, association or youth organization).	**.83**
Participated in a leadership group or committee working on issues related to ethnicity, heritage and/or discrimination (e.g., student council, association or youth organization).	**.81**
Attended a protest on an issue related to ethnicity, heritage and/or discrimination.	**.82**
Invited someone to a meeting or protest related to ethnicity, heritage and/or discrimination.	**.87**
Inspired others to work on issues related to ethnicity, heritage and/or discrimination.	**.77**
Researched/investigated issues or social problems regarding ethnicity, heritage and/or discrimination in my community.	**.60**
Spoken out on social media about issues related to ethnicity, heritage and/or discrimination.	**.81**
Donated money to an initiative that works on issues related to ethnicity, heritage and/or discrimination (e.g., charity or other type of organization).	**.62**
Signed a petition about an issues regarding ethnicity, heritage and/or discrimination.	**.79**
**Deleted items**	
Challenged or checked myself before using a racist slur or making a racist joke.[a]	
Contacted the media (e.g., newspaper, TV-broadcaster, website) when you have seen something that is racist.[b]	
Contacted an elected official about an issue related to ethnicity, heritage and/or discrimination (e.g., minister, member of national parliament, member of local parliament, alderman)[b]	
Organized your own project on an issue related to ethnicity, heritage and/or discrimination.[b]	
Actively participated in a political party or political youth organization to work on issues regarding ethnicity, heritage and/or discrimination.[b]	
Reported (online) racism to a discrimination hotline.[b]	

Note. [a] = factor loading below .40, [b] = low frequency variable.

Table S4. Model fit indices for 1 to 6 factor solutions on critical action measure (EFA)

	χ^2 (df)	*p*	RMSEA	SRMR	CFI	TLI
1-factor	279.80 (135)	<.001	0.06	0.14	0.92	0.91
2-factor	179.33 (118)	<.001	0.04	0.10	0.97	0.96
3-factor	127.98 (102)	.042	0.03	0.09	0.99	0.98
4-factor	97.31 (87)	.211	0.02	0.07	0.99	0.99
5-factor	70.37 (73)	.566	0.00	0.05	1.00	1.00
6-factor	N/A					

Eigenvalues were above one for the first five factors (7.69, 1.85, 1.44, 1.15 and 1.13 respectively), suggesting that the 6-factor model is not appropriate. The 6-factor model additionally did not converge. Model fit indices generally indicated that the 1-factor solution already had acceptable fit (RMSEA, CFI and TLI), and that fit got better for the solutions with more factors (Table S4). SRMR only became acceptable starting at the 4-factor solution. However, the 2- to 5-factor solutions all had at least one factor which did not have a minimum of three indicators loading .40 or higher (after removing indicators loading .40 or higher on multiple indicators and assigning other cross-loading indicators to the factor they load highest on). Therefore, it seems that the 1-factor solution model actually fits best, and that no reliable multiple-factor model can be fitted to the data in order to use subfactors in exploratory analyses.

Supplementary Materials 3: EFA on parental ethnic-racial socialization

An exploratory factor analysis (EFA) was run on the parental ethnic-racial socialization (ERS) measure (Hughes & Chen, 1997; Hughes & Johnson, 2001; Pahlke et al., 2012). The EFA was conducted using Mplus 8.10, allowing for correlations between factors by using Geoming rotating and addressing missing data under Full Information Maximum Likelihood conditions (FIML). Between 1 and 8 factor-solutions were evaluated. The number of factors is again determined based on eigenvalues, model fit indices, and interpretability of the factors. Furthermore, factors should have at least three indicators and indicators were considered salient if factor loadings were above .40. In case of cross-loadings, items were retained to the factor it loads .40 or higher on. If items loaded above .40 on multiple items, they were deleted in order to retain a simple structure.

Eigenvalues were above one for the first five factors (9.76, 2.50, 1.56, 1.36, and 1.04, respectively), suggesting that the 6-, 7-, and 8-factor solutions were not suited. The 8-factor model additionally did not converge. All model fit indices indicate that the 1-factor solution is not an acceptable fit (RMSEA and SRMR >.08, CFI and TLI <.90, see Table S5). For the 2-, 3- and 4-factor solution, SRMR does indicate an acceptable fit, but the other indices do not, whereas for the 5-factor solution, most indices indicate an acceptable fit (RMSEA, SRMR, CFI), with the exception of TLI.

Table S5. Model fit indices for 1 to 8 factor solutions on parental ERS (EFA)

	χ^2 (df)	*p*	RMSEA	SRMR	CFI	TLI
1-factor	1573.69 (275)	<.001	0.13	0.10	0.70	0.67
2-factor	1198.67 (251)	<.001	0.11	0.07	0.77	0.73
3-factor	927.74 (228)	<.001	0.10	0.05	0.84	0.78
4-factor	678.70 (206)	<.001	0.09	0.04	0.89	0.84
5-factor	497.95 (185)	<.001	0.08	0.03	0.93	0.88
6-factor	406.65 (165)	<.001	0.07	0.03	0.94	0.90
7-factor	291.53 (146)	<.001	0.06	0.03	0.97	0.93
8-factor	N/A					

However, the 3-, 5-, 6-, and 7-factor solutions all had at least one factor which did not have a minimum of three indicators loading .40 or higher (after removing indicators loading .40 or higher on multiple indicators and assigning other cross-loading indicators to the factor they load highest on). Therefore, the 4-factor solution is deemed most appropriate combining information on model fit indices, salience of factors, and interpretability.

The factor loadings (Table S6) show that factor 1 consists of 6 items, factor 2 consists of 4 items, factor 3 consists of 8 items, and factor 4 consists of 4 items. The items in factor 1 mostly stem from the original egalitarianism subscale, and consist of messages stressing the importance of seeing and treating people of all different backgrounds equally. This factor is therefore also referred to as *egalitarianism* in the present study. All items in factor 2 were previously included in the subscale *history of other groups*, and is also named as such in the present study. Most of the items in factor 3 tap into messages about *discrimination against other groups*. The items in factor 4 reflect messages rejecting intergroup interactions and stressing reverse racism (i.e., against own group in this study means white people). Therefore, this factor reflects *negative intergroup socialization*.

For this study, we are interested particularly in forms of socialization that can be defined as positive color-conscious socialization practices. Therefore, factors 1, 2, and 3 will be included in the main analyses of the present study, whereas factor 4 will not.

Table S6. Factor loadings for the 4-factor solution of parental ERS (EFA)

	F1	F2	F3	F4
told you that you should try to make friends with people of all ethnicities and heritages.	**.44**	.16	.07	.21
told you about the importance of getting along with people of all ethnicities and heritages.	**.47**	.12	.22	.18
told you people of all ethnicities and heritages have an equal chance in life.	**.57**	.00	-.01	.17
told you it is important to appreciate people of all ethnicities and heritages.	**.70**	-.01	.23	-.02
told you people are equal, regardless of their ethnicity or heritage.	**.65**	-.08	.31	-.08
told you about important people in the history of other ethnic or heritage groups.	.09	**.76**	.00	-.03
told you about the history of other ethnic or heritage groups in our country.	-.06	**.83**	.11	-.03
taught you about the history or traditions of other ethnic or heritage groups.	.00	**.69**	.11	-.01
read books about the history or traditions of different ethnic and heritage groups, other than your own.	-.02	**.67**	-.02	.10
told you it is best to have friends from the same ethnic or heritage group as you are.	-.17	.05	-.02	**.77**
told you people of different ethnicities and heritages have different values and beliefs.	.04	-.07	**.41**	.34
told you it is a bad idea to marry someone from a different ethnic or heritage group.	.32	-.07	.12	**.71**
told you about the possibility that some people might treat you badly or unfairly because of ethnicity or heritage.	.04	.33	-.06	**.45**
told you about discrimination or prejudice against your ethnic or heritage group.[a]	.03	.30	.02	.35
told you society is fair to all ethnicities and heritages.	.37	.00	-.22	**.58**
told you sometimes people are treated badly just because of their ethnicity or heritage.	.00	.05	**.79**	.03
told you society is not always fair to all ethnicities and heritages.	.01	.00	**.79**	-.04
told you other ethnic or heritage groups are just as trustworthy as people of your own ethnic or heritage group.	**.47**	.16	.17	-.01
told you about the discrimination people from other ethnic or heritage groups have experienced in the past.	.13	.34	**.42**	-.03
told you about discrimination or prejudice against other ethnic or heritage groups.	.08	.16	**.64**	-.02
told you that people from other ethnic or heritage groups are sometimes still discriminated against because of their ethnicity or heritage.	.00	.16	**.78**	-.02
told you that in the past, people from other ethnic or heritage groups were discriminated against because of their ethnicity or heritage.	.04	.18	**.64**	.01
told you that people of your ethnic or heritage group have better opportunities than people of other ethnic or heritage groups.	-.07	-.04	**.63**	.08
told you about something unfair that he/she witnessed that was due to ethnic discrimination against another ethnic or heritage group.[a]	.10	.23	.38	.10
told you about something he/she saw that showed poor treatment of different ethnic or heritage groups, other than your own.[a]	.02	.28	.39	.11

Note. [a] = factor loadings were below .40. **Bold** = indicator of this factor.
